# Bias dependence in statistical random telegraph noise analysis based on nanoscale CMOS ring oscillators

**DOI:** 10.1007/s00502-023-01197-3

**Published:** 2023-12-21

**Authors:** Semih Ramazanoglu, Alicja Michalowska-Forsyth, Bernd Deutschmann

**Affiliations:** Institute of Electronics (IFE), Graz University of Technology, Inffeldgasse 12/I, 8010 Graz, Austria

**Keywords:** Random telegraph noise, RTN, Jitter, Ring oscillator, Oxide trap, Telegrafenrauschen, RTN, Jitter, Ringoszillator, Oxid-Falle

## Abstract

Random Telegraph Noise (RTN) is one of the major reliability concerns in nanoscale complementary metal-oxide semiconductor (CMOS) technologies. In this paper, we discuss the characterization of RTN in 40 nm CMOS technology using Ring Oscillators (ROSCs). We used different types of ROSCs to study the temporal and spectral characteristics of the RTN. We conducted measurements on one of the arrays with 128 identical ROSC cells. These results enabled statistical characterization of the RTN amplitude strength and its frequency characteristics in different supply voltage variations from 0.5 V to 0.7 V. At power supply of 0.65 V, dominant and observable RTN amplitude above 0.37% $\Delta f/f_{\text{mean}}$ is found in 60% of cells in the array. Further, the capture and emission time constant $\tau_{e//c}$ can be extracted from the measurements, the values observed ranging from 0.2 $\upmu$s to 10 ms.

## Abstract (de)

Das zufällige Telegrafenrauschen (RTN) stellt die Zuverlässigkeit von komplementären Metall-Oxid-Halbleiter-Technologien (CMOS) vor große Herausforderungen. In diesem Beitrag diskutieren wir die Charakterisierung von RTN in 40 nm CMOS-Technologie unter Verwendung von Ringoszillatoren (ROSCs). Verschiedene Arten von ROSCs wurden verwendet, um die zeitlichen und spektralen Eigenschaften des RTN anhand eines Arrays mit 128 identischen ROSC-Zellen zu untersuchen. Diese Ergebnisse ermöglichten die statistische Charakterisierung der RTN-Amplitude und ihrer Frequenzcharakteristiken bei Versorgungsspannungsvariationen von 0,5 V bis 0,7 V. Bei einer Versorgungsspannung von 0,65 V wird in 60 % der Zellen des Arrays eine dominante und beobachtbare RTN-Amplitude über 0,37 % $\Delta f/f_{\text{mean}}$ gefunden. Darüber hinaus kann die Einfang- und Emissionszeitkonstante $\tau_{e//c}$ aus den Messungen extrahiert werden, wobei die beobachteten Werte zwischen 0,2 ${\upmu}$s und 10 ms liegen.

## Introduction

The size of CMOS transistors has been reduced due to significant progress in the development of nanoscale technologies [1]. With this development, the RTN phenomenon has become one of the major low-frequency noise sources. RTN is observed as temporal fluctuations in the threshold voltage ($V_{\text{th}}$) and, consequently, the drain current. It is a known fact that RTN has inevitable impacts on digital circuits [2].

Furthermore, the RTN phenomenon has been studied empirically to understand how oxide charge trapping and detrapping mechanisms affect CMOS circuits. Most studies on RTN have focused on the characterisation and modelling of a single MOSFET device, as in [3, 4], and [5].

Besides single device methodologies, a considerable amount of studies have investigated the impact of RTN on time-dependent IC circuits. Influences of jitter and phase noise on the circuit performance were analyzed in [6]. Subsequently, to analyze the RTN’s effects on MOSFET devices during switching operations, studies [7, 8] have provided remarkable knowledge of the RTN from ROSC based analysis. Since the key parameter of ROSCs is the oscillation frequency, by narrowing the observation to the frequency difference between two ROSCs’ signals, a high measurement resolution can be achieved by this “dual ROSC method” [9]. From the design perspective, focusing on the simulation tools and techniques is a practical approach. Consequently, Verilog AMS model [10] accounting for transient gate-voltage fluctuations was proposed. Regarding simulation methods, an analytical model [11] can be applied to account for $V_{\text{th}}$ fluctuations.

Previous studies have significantly contributed to the knowledge of RTN amplitude in single CMOS devices and ROSCs, providing valuable insights into this field. However, much remains to be addressed. The RTN-amplitude for $\Delta f/f_{\text{mean}}$ mean serves as a measure of the magnitude of the RTN-induced frequency fluctuations in relation to its typical value resulting from the presence of noise events. The combined time constant, $\tau_{e//c}$, encompasses both the capture $\tau_{c}$ and emission $\tau_{e}$ duration, and affects the overall unique characteristics of the RTN. Specifically, we investigated the statistical analysis and bias dependence of the RTN in an array consisting of 128 cells of 5 stages of differential inverter-based ROSCs with a low slew rate. Our analysis aimed to comprehend the RTN characteristics and variability of $\Delta f/f_{\text{mean}}$ and $\tau_{e//c}$ for different types of ROSCs. Primary statistical results have been reported for differential-inverter-based ROSCs array to indicate the supply voltage dependence of RTN activation in dynamic circuits by utilizing ROSCs, as similarly shown by the single-device bias dependency of RTN [12]. Our findings in the statistical analysis confirm the bias dependence trends are related to defect localization within the energy band-gap. The measurements show a representative picture on the density of defects at different biasing conditions. This is an extended version of a preliminary conference work that was presented in Austrochip 2023 [13].

## Random telegraph noise in dynamic circuits

The CMOS fabrication process unavoidably introduces gate oxide traps, with high manufacturing effort to minimize their density. The measurable characteristics of the traps are their charge capture and emission times, and the subsequent change in the drain current amplitude. A further implication is the effective $V_{\text{th}}$ fluctuation caused by the trapping-detrapping mechanism. Accordingly, it is commonly accepted [7, 14] that the $V_{\text{th}}$ variation ($\Delta V_{\text{th}}$), due to one electron charge trapped at the $\text{SiO}_{\text{2}}$-Si interface is described by (1) and applies also to RTN analysis, where $q$ is the elementary charge carrier, $C_{\text{ox}}$ is the gate oxide capacitance. $W$ and $L$ refer to the width and length of the MOSFET device.

1 $$\Delta V_{\text{th}}=\frac{q}{C_{\text{ox}}WL}$$

**Fig. 1:**
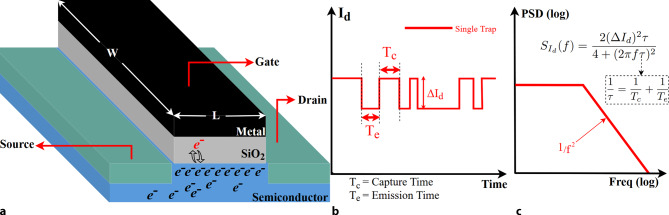
RTN behavior and Oxide Trap Mechanism. **a** Shows the schematic 3D-Illustration of Oxide Trap mechanism for single trap. **b** The time domain illustration of drain current ($I_{d}$) with single trapping mechanism. **c** The illustration of a RTN spectrum in log-log frequency domain of $I_{d}$ in **b**

A 3D-Illustration of the oxide trap mechanism can be seen in Fig. 1a. In relation to this, Fig. 1b shows the single oxide trap which affects drain-current $I_{d}$ transient behaviors that are caused by $\Delta V_{\text{th}}$. Fig. 1c demonstrates power spectral density (PSD) of characteristic Lorentzian shape of RTN-induced $\Delta I_{d}$ in the frequency domain in Fig. 1b. The fundamental formula of noise PSD [15] is indicated in Fig. 1c.

**Fig. 2:**
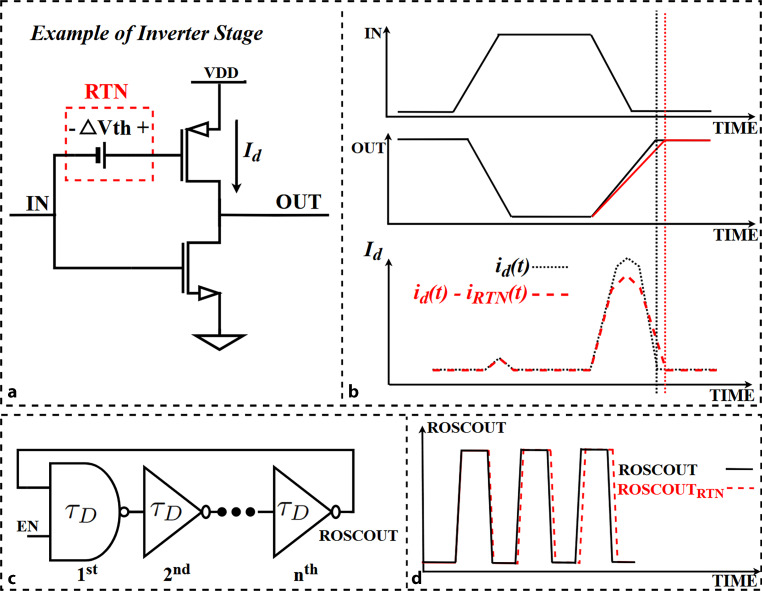
Schematic illustration of **a** an inverter with RTN representative module, **b** RTN impact on output and $I_{d}$ of driving transistor of an inverter in time domain, **c** a basic inverter-chain ROSC and **d** the RTN impact on the output signal, resulting in frequency shift

During normal operation, if RTN amplitude $\Delta V_{\text{th}}$ dominates over the other noise sources in the delay stage as modeled in Fig. 2a, the RTN-induced time delay pattern of IN-OUT signals must also correspond to the temporal RTN activity as shown in Fig. 2b. A basic ROSC circuit and output signal are illustrated as a transient shift of the “ROSCOUT” signal in Fig. 2c and d. The fundamental formula of the frequency of a ring oscillator is given by (2). It includes $n$ identical inverter stages and the total delay time ($\tau_{D}$) of a single inverter stage which consists of rise-edge and fall-edge delay time [16].

In (3) [17], $\tau_{D}$ represents the time delay by each stage of a ROSC. $C_{\text{Load}}$ refers to the load capacitance, $V_{DD}$ represents the supply voltage, $W$ and $L$ are the width and length of the driving transistor (pull up or pull down), respectively, $\mu$ represents the carrier mobility. $\tau_{D}$ is the nominal value that is further contaminated with Gaussian noise and with RTN-induced time delay. In the model that we use, RTN results in the effective $V_{\text{th}}$ shift, which in consequence influences the inverter delay time.

2 $$f_{\text{ROSC}}=\frac{1}{2n\tau_{D}}$$

3 $$\tau_{D}=\frac{C_{\text{Load}}V_{DD}}{\frac{W}{L}\mu C_{\text{ox}}(V_{DD}+V_{\text{th}})^{2}}$$

## Methods and measurement results

**Fig. 3:**
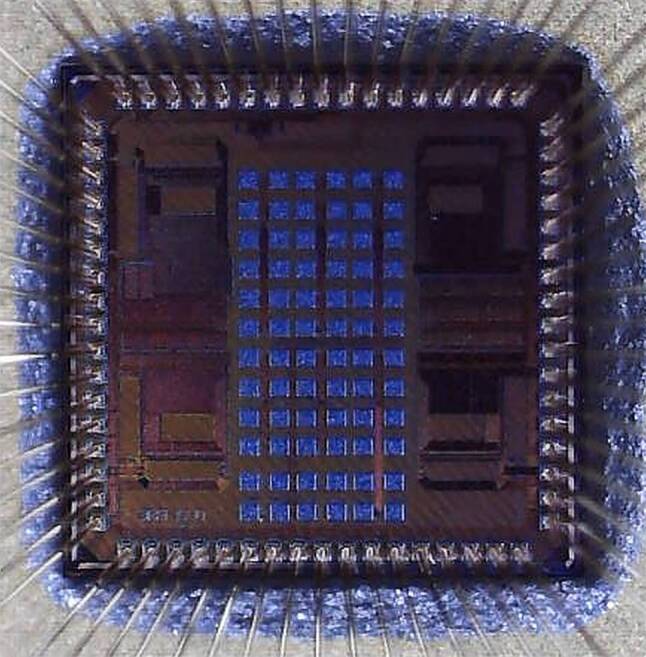
Bonded die of SIRENS-40 CMOS chip in 40 nm (1.7 mm $\times$ 1.7 mm)

The main goal of the presented studies is a statistical observation of the RTN influence on ROSCs under different supply voltage variations. We have used ROSC circuit architectures of different slew rates to monitor how it influences frequency fluctuations due to RTN. Testchip in 40 nm bulk CMOS with die area 1.7 mm $\times$ 1.7 mm is illustrated in Fig. 3. Out of a broad family of ROSCs, those discussed in this paper are comparable in measurable operation frequencies on bonded dies in ceramic package. The two ROSCs are a basic inverter-chain and shunt-capacitor differential inverter as shown in Fig. 5a and b. We conducted an in-depth examination of four distinct types of ROSCs, with a particular emphasis on the statistical analysis of the differential inverter based ROSC structure. Notably, the additional supply noise rejection in the differential structure is higher than in the basic inverter structure. However, this information is very likely influenced by the upper and lower frequency limitations of noise measurement systems. Undoubtedly, we found that ROSC method is only feasible when the oscillation frequency factor is at least 10 times higher than cut-off frequency of the RTN’s Lorentzian spectrum (i.e., $f_{\text{ROSC}}\gg f_{\text{RTN}}$). Previous studies [2, 10] have shown that the RTN-time scale of capture and emission is in the range of a few seconds to microseconds, corresponding to a maximum frequency range of MHz.

**Fig. 4:**
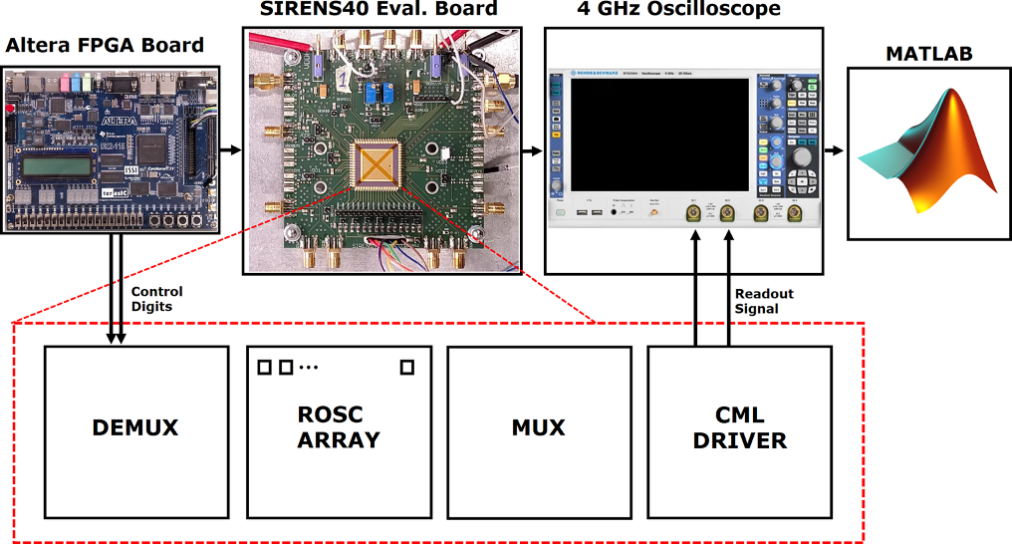
Measurement Setup

The measurement setup employed an Altera Board in the form of a digital controller, a device under test (DUT) utilizing the SIRENS40 Evaluation Board, a 4 GHz oscilloscope with a 20 G sampling rate, and MATLAB software for characterization, as shown in Fig. 4. The microcontroller generated parallel bits to manage the demultiplexing of the chip and selected individual ROSCs from an array of 128 ROSCs. The output signal waveform of the current-mode logic (CML) Driver was examined using the oscilloscope. Following multiple data acquisitions, all signals were analyzed using MATLAB to determine the frequency as a function of time ($f(t)$). Additionally, the frequency domain characteristics of the RTN-induced $f(t)$ waveform were analyzed using MATLAB.

**Fig. 5:**
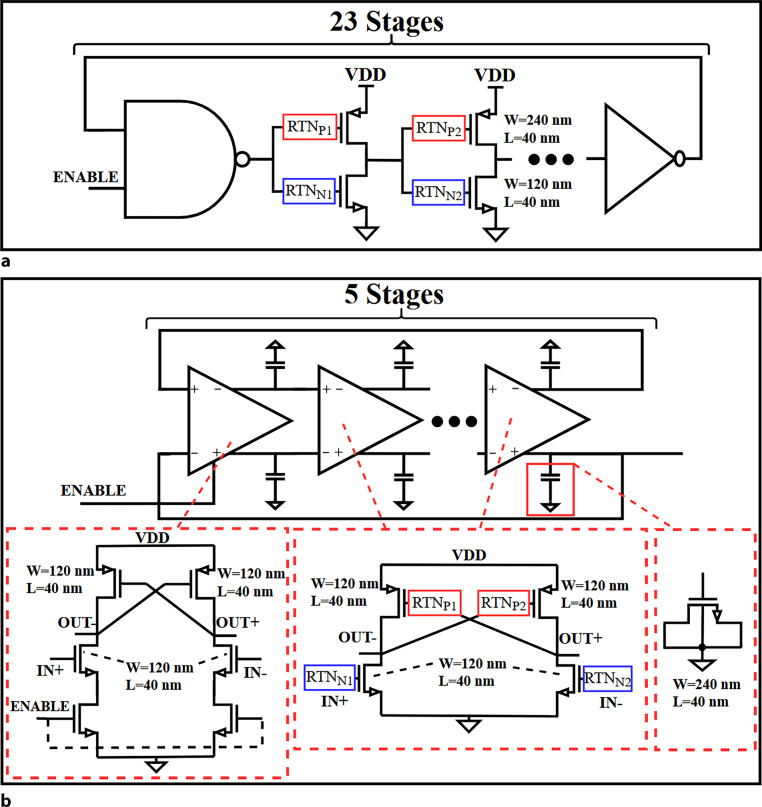
Schematic view of **a** a basic inverter-chain based, **b** and a shunt MOSFET-capacitor integrated differential inverter-based ROSCs

**Fig. 6:**
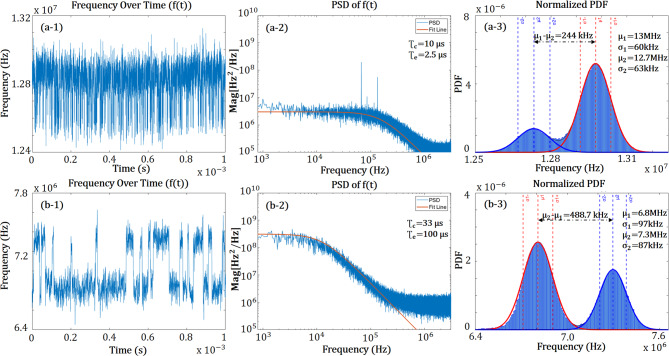
RTN behavior of (a-1) frequency over time $f(t)$, (a-2) PSD of RTN-induced frequency fluctuation and (a-3) PDF of $f(t)$ of inverter-chain ROSC in Fig. 5a. RTN behavior of (b-1) frequency over time $f(t)$, (b-2) PSD of RTN-induced frequency fluctuation (b-3) PDF of $f(t)$ of Differential Inverter ROSC in Fig. 5b. These are results from measuring one ROSC of each type; time of a single observation 1 ms, sampling rate 20 G samples per second, 10 repeated acquisitions

In Fig. 6, the combination of the three types of plots gives a thorough comparison of the RTN-induced frequency variations for the two circuits (a and b) from Fig. 5. Furthermore, Fig. 6a‑1 and b‑1 illustrate the erratic nature of the RTN, indicating its random and unpredictable behavior over time. Fig. 6a‑2 and b‑2 shows the measurement in the frequency domain, overlapped with fitted ideal Lorentzian PSD of the frequency fluctuations, which emphasizes the typical PSD shape of the RTN. However, it is important to note that phase-noise-induced frequency fluctuations have an impact on the PSD shape of the RTN of ROSCs at higher frequencies (background noise level). The Probability Density Function (PDF) of time domain samples of frequency shown in Fig. 6a‑3 and b‑3, enables analysis of the system characteristics: RTN amplitude, Gaussian phase noise, rms-jitter, relation between mean RTN capture and emission times.

**Table 1:** Measurement Results of Single Level RTN-induced $\Delta f/f_{\text{mean}}$ (%) for (a and b) circuits in the Fig. 5 with different VDD

VDD (V)	0.50	0.55	0.60	0.65	0.70	0.75	0.80	0.85	0.90	1.00
Circuit (a)	1.88	1.63	1.41	1.10	0.93	0.73	0.31	0.17	0.12	0.04
Circuit (b)	7.18	5.93	3.93	3.65	2.32	0.48	0.20	0.19	0.11	0.05

In Table 1, the results present the detectable impact of RTN through the multiple measurements of $\Delta f/f_{\text{mean}}(\%)$ in two circuits, denoted as a and b in Fig. 5. The circuits were examined under different supply voltages (VDD) ranging from 0.5 V to 1 V. The recorded measurement pattern suggests the effect of bias dependence on the RTN phenomenon reported in detail in [12]. Initially, Circuit (b) showcased higher frequency fluctuation at lower VDD values (0.5–0.7 V), indicating more dominant detectable impact of RTN amplitude than Circuit (a). ROSCs with lower number of stages are more effective to analyze RTN impacts. Since these are results on a random samples and not reflecting the statistical picture, the observation can be attributed to different characteristics of RTN levels in the two different circuits under test. However, according to simulation based analysis, it is Circuit (b) where a stronger influence of RTN on frequency fluctuation is expected (assuming RTN of identical amplitudes appearing in a single MOSFET in each of these circuits). At higher VDD values (0.9–1 V), both circuits exhibited noticeably reduced frequency fluctuations due to RTN. Also, the transient phase noise became more dominant than the detectable RTN-induced frequency jumps making RTN more difficult to detect. The preliminary observation suggests that the method of power supply sweep is well suited to study the fundamental mechanisms of RTN in the process node used in this study.

Interestingly, already these preliminary results are consistent with transient noise simulations with integrated $\Delta V_{\text{th}}$ module in the design environment, where Circuit (b) had 3.1 to 3.8 times higher $\Delta f/f$ than Circuit (a). These simulation results cover power supply level of 0.5 V and 0.6 V. They also assume RTN-induced $V_{\text{th}}$ fluctuations of identical amplitude and in only one MOSFET in each circuit. Meanwhile the absolute Gaussian phase noise (due to flicker and thermal noise) is only 1.7 to 2 times higher in Circuit (b) than in Circuit (a). These numbers signify that the RTN-related peaks in PDF are easier distinguishable in Circuit (b) if we deal with identical RTN amplitudes in a single noisy transistor. These results do not account for power supply noise, which is well attenuated in the differential Circuit (b). Potential occurrence of multiple RTN levels will be therefore also easier to identify. An experimental conclusion on the Circuit (a) and Circuit (b) comparison requires measurements on a large number of circuits for a broad statistical coverage.

## Statistical results

**Fig. 7:**
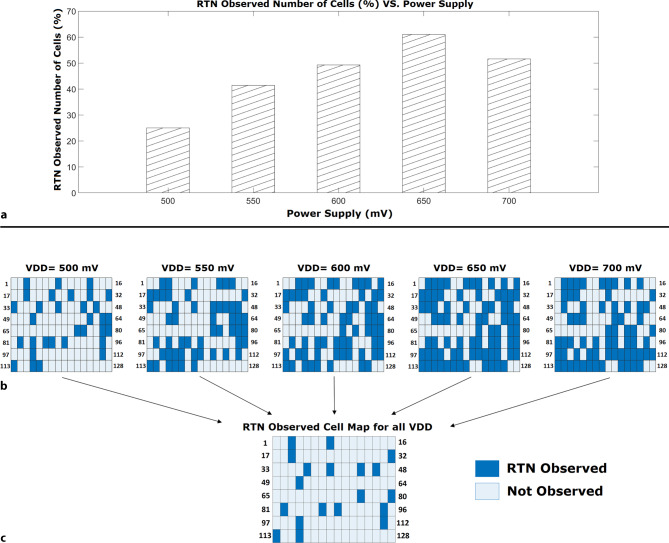
**a** RTN Observed Statistics in $(\%)$ and **b** Cell-Map for differential inverter based structure ROSCs array for each VDD level varying from 0.5 V to 0.7 V and **c** active RTN Observed map for all VDD levels

The statistical analysis presented in this work was carried out on the differential ROSC in Fig. 5. This choice was driven by the promising simulation results for this array also seen in the preliminary measurements in Fig. 6. Furthermore, the frequency range in this array (ranging from 1 MHz to 1 GHz) made it possible to measure it in a ceramic package with an oscilloscope. Fig. 7 provides a clear and illustrative statistics of the measurements for RTN occurrence in a differential structure array. Following output voltage waveform measurements, the primary microchip’s supply voltage-related RTN behavior in the ROSCs was statistically characterized. We found that the variability in voltage spans and the behavior of oxide traps, especially in terms of required trap activation voltage potential. The trap’s activation and deactivation by VDD variations had a significant impact on statistics. Analysis of 2D-Map illustrated Fig. 7 revealed discrepancies in the voltage levels. The percentage of ROSCs in which RTN was detected in the array varied from 0.5 V to 0.7 V.

From statistical perspective, experimental results indicate variability in both the scope of bias dependency and the peak $\Delta f/f_{\text{mean}}$ (%) values. The dataset in Fig. 7a outlines the correlation between VDD values and the percentage of RTN observed number of ROSCs in the array. The discrete VDD values plotted on the horizontal axis range from 0.5 V to 0.7 V in 50 mV increments. The vertical axis represents RTN observed number of cells in (%). From this dataset, for the VDD of 0.5 V, the RTN occurrence is recorded at 25%. As VDD increases to 0.55 V, RTN occurrence elevates to 41%. This trend persists with VDD values of 0.6 V and 0.65 V, where RTN occurrences are 49% and 60%, respectively. At 0.7 V, the number of RTN observed cells reduces to 51%.

Fig. 7b presents a 2D-Map of RTN observed ROSC cells in relation to their location in the array for varying VDD form 0.5 V to 0.7 V. Because of the bias dependence, there are ROSC cells with observable RTN only at some VDD conditions while at the other levels. RTN clean circuits without observable RTN are labelled as “Not Observed” in Fig. 7b. Finally there is a group where a dominant RTN was recorded at each of the analysed VDD levels. This is a particularly interesting group from the RTN bias dependence analysis point of view. Fig. 7c shows active RTN-Observed cells 2D-Map for all VDD levels.

**Fig. 8:**
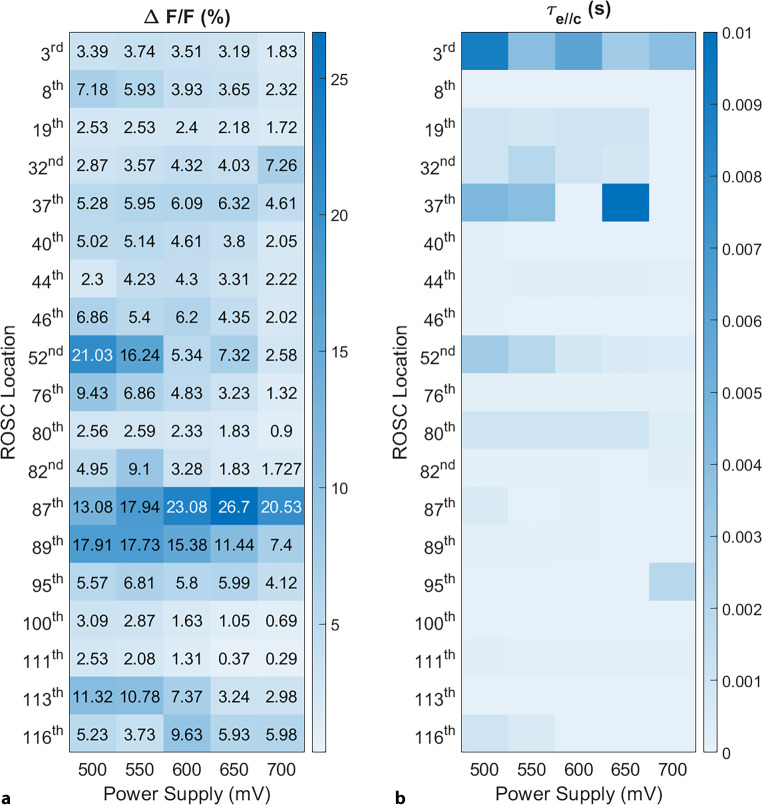
**a** Normalized $\Delta f/f_{\text{mean}}$(%) and **b** capture and emission time constant for ROSC cells where the RTN was detected at each of the measured VDD levels from 0.5 V to 0.7 V

Finally, Fig. 8a shows quantitative results of $\Delta f/f_{\text{mean}}$ (%) due to RTN in these cells had detectable RTN was observed at each of the measured VDD levels. Each table row denotes a specific oscillator cells, marked by its unique ROSC number related to its location in the array. The analysis of the $\tau_{e//c}$ values across VDD ranging from 0.5 V to 0.7 V is presented in Fig. 8b. It was found that the $\tau_{e//c}$ constants are varying for every VDD variations. Further, the observed range of $\tau_{e//c}$ variables is from 0.2 $\mu s$ to 10 $ms$. However, there is no observable correlation between the $\tau_{e//c}$ values and the amplitude of the variable $\Delta f/f_{\text{mean}}$ (%).

**Fig. 9:**
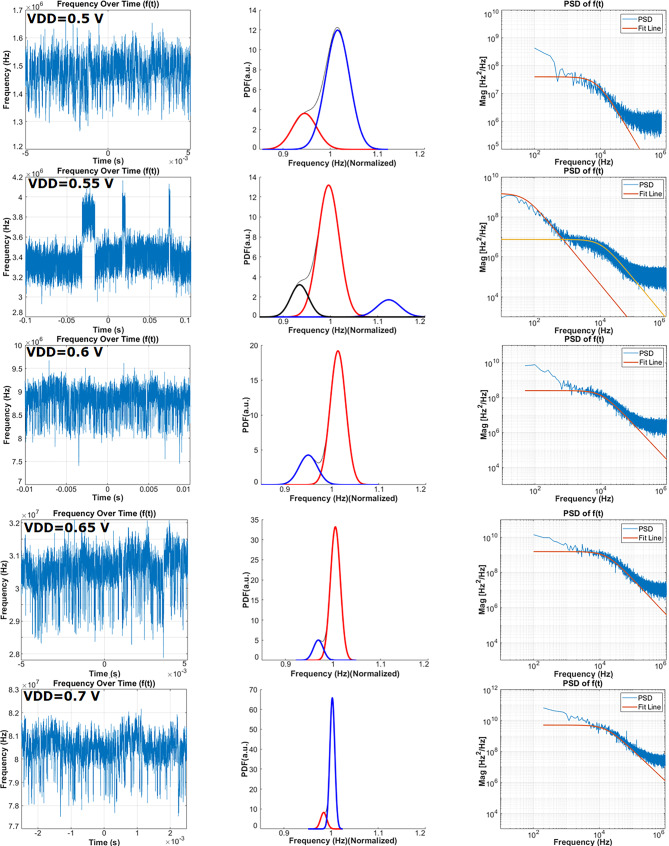
Single and double traps observable with the ROSC-based RTN analysis method with characteristics changing as a function of the power supply with VDD ranging from 0.5 V to 0.7 V with 50 mV increments. *Left:* Time domain frequency fluctuations. *Middle*: probability density function of time domain data. *Right:* Power spectral density of temporal frequency fluctuations

In analogy to [12], the characterization of individual RTN devices and their bias dependency curves are influenced by power supply voltage bias variations in operating frequency. For example, consider the oscillator whose binary control digit enables the 87th ROSC in the array in Fig. 8a. This oscillator demonstrates notable RTN-induced frequency shifts in terms of VDD. In contrast, configurations like the 89th ROSC exhibit consistent trends across the VDD increment, activating and deactivating different traps at the $\text{SiO}_{\text{2}}$-Si interface. The primary cause of this phenomenon is changes in electric field required to activate the traps at specific voltage thresholds. For instance, in our examination of the double traps, there are two traps named Trap‑1 and Trap‑2. Double traps can be seen in Fig. 9. Trap‑1 was activated for VDD varying from 0.5 V to 0.7 V. Trap‑2 was only activated at VDD level 0.55 V. Fig. 9 shows more details for RTN characteristics in time and frequency domain. Also, normalized distribution of $f(t)$ can be seen in Fig. 9. Similarly, another case Trap‑1 could be active from 0.5 V to 0.6 V, while Trap‑2 was inactive. When the VDD increased from 0.6 V to 0.7 V, Trap‑2 was activated, Trap‑1 was inactive.

It is essential to note that the nature of RTN is a complex phenomenon, as demonstrated in single device investigation [12], and yet there remains much to be understood about this topic in dynamic circuits. Our findings support this complexity and highlight the strong cell-to-cell variability and bias dependence profile of RTN. The observation of RTN in dynamic measurements presents a new path for further investigation, particularly when analyzed in conjunction with characterization under varying operating conditions, such as VDD and possibly temperature.

Furthermore, the method used in this ROSC study to characterize RTN, including the direct measurement of packaged IC samples with on-chip multiplexing and within the MHz to GHz frequency range, can be readily implemented in other process nodes and flavors. There are additional avenues to advance the ROSC-based RTN system: (A) Direct bonding of the chip die to the PCB enables faster ROSC characterization, reducing the number of contributing transistors and the maximum observable RTN frequency shifts. (B) Enhancing data acquisition can reveal more observable trapping/detrapping activity for RTN-induced frequency over a longer time window. This improvement aims to obtain more statistical details from a single trap and to detect traps with very long time constants. In larger MOSFETs, the low-frequency noise closely resembles the well-known 1/f noise behavior, with dominant RTN occurrences being rare. However, our system is especially pertinent for a better understanding of random trapping/detrapping effects in small MOSFETs within dynamic circuits. Here, instantaneous time-delay fluctuations can be critically impactful. Implementing this characterization method in different process nodes and flavors can offer a more profound understanding of RTN and its influence on circuit performance.

## Conclusion

We explored the statistical characteristics of RTN in the 40 nm CMOS process with two types of ROSCs. We analyzed the statistical data of 128 ROSCs of each type under different operating voltage conditions. Our results show that RTN can cause significant frequency shifts in the ROSCs, and the RTN amplitude $\Delta f/f_{\text{mean}}$ above 0.37% can be observed in 60% of the cells in the array at power supply levels ranging from 0.5 V to 0.7 V. In addition, we extracted the capture and emission time constants $\tau_{e//c}$ from the measurements and observations, with the values observed ranging from 0.2 $\upmu$s to 10 ms. Our findings provide valuable insights into the complex nature of RTN in the 40 nm CMOS process node under switching devices, which is crucial for developing strategies to minimize the impact of RTN on integrated circuits.
